# To See the Telemedicine Forest for Its Multicultural Trees. Comment on: In‐Home Remote Assessment of the MDS‐UPDRS Part III: Multicultural Development and Validation of a Guide for Patients

**DOI:** 10.1002/mdc3.70073

**Published:** 2025-05-13

**Authors:** Fabiana de Moraes Goraieb, Daniel Teixeira‐dos‐Santos, Artur Francisco Schumacher Schuh, Pedro Renato de Paula Brandão, Laura Silveira‐Moriyama

**Affiliations:** ^1^ Department of Neurology University of Campinas (Unicamp) Campinas Brazil; ^2^ Department of Neurology Hospital de Clínicas de Porto Alegre Porto Alegre Brazil; ^3^ Department of Neurology Santa Casa de Misericórdia de Porto Alegre Porto Alegre Brazil; ^4^ School of Medicine University of Brasília Brasília Brazil; ^5^ Research and Education Institute, Hospital Sírio‐Libanês Brasília Brazil; ^6^ Education Unit University College London Institute of Neurology, University College London London United Kingdom

**Keywords:** telemedicine, movement disorders, Brazil, Parkinson's disease

We would like to thank Tosin et al.^1^ for the innovative multicultural development of a guide for patients undergoing the Movement Disorders Society Unified Parkinson's Disease Rating Scale (MDS‐UPDRS) Part III via telemedicine and recognize its potential for global application. We further emphasize the importance of taking into consideration socioeconomic factors related to telemedicine.

Their guide, originally prepared in English and later translated into Spanish, Portuguese, and Chinese, effectively used minimal text and clear visuals to facilitate adaptation and global dissemination. Although in their study 80% of patients had “associate, graduate, or bachelor's degree,” they highlighted those Brazilian patients had significantly lower educational levels (*P* = 0.02). This observation aligns with findings from 3 independent real‐world telemedicine studies conducted in southern and southeastern Brazil, regions considered relatively affluent compared to the country as a whole. Two studies assessing telemedicine feasibility in southern Brazil^2^, ^3^ found only 17.4% (12 of 69)^2^ and 17.6% (6 of 34)^3^ of the patients or caregivers interviewed had a bachelor's degree or higher. Similarly, our sample of Parkinson's disease from Campinas (southwest Brazil) revealed a comparable rate of 18.7% (14 of 75) with such degrees. In 1 study^2^ higher educational and socioeconomic levels were associated with greater telemedicine adherence (*P* = 0.01 and *P* = 0.03, respectively).^2^

Despite challenges, the economic impact of offering telemedicine might be even more relevant in limited‐resource settings. In our sample (n = 95), patients traveled on average 58.2 min (range: 9–249; standard deviation [SD] ± 55.45) to reach the in‐person consultation, whereas in another sample of 130 parkinsonism patients from the same group from southern Brazil, 69 individuals who responded reported spending 91.30 min (SD ± 87.7).^2^ Telemedicine leads to a reduced need (*P* = 0.003, Fisher) for family members to help patients attend telemedicine visits (57.1%, 32/56) compared to in‐person (83.9%, 47/56) visits in our study. Direct savings with transportation and meals from attending telemedicine as opposed to in‐person visits were on average US$12.07 (range: US$0–59.63; SD ± 11.91, N = 56) per visit, about 5.47% (range: 0–30.40; SD ± 6.39, N = 55) of the monthly income (average per‐capita monthly income of US$331.69 per month, US$51.62–1109.03; SD ± 250.58, N = 55). In economic scenarios of scarcity like ours, small proportions of a monthly income might correspond to essential needs of the family not being met that month. In addition, in‐person costs are grossly underestimated because many patients use government‐funded transportation, which is reflected in the governmental health‐budget.

Educational and economic levels might be difficult to change, but modest interventions like this guide can enhance adherence to telemedicine. Simple bureaucratic hurdles like outdated or incorrect contact information prevented about a third of patients from participating in our studies (44 in 130 of parkinsonian patients in 1 study^2^ and 45 in 140 movement disorders patients in our sample).

We believe that facilitating telemedicine in movement disorders can greatly enhance the care of patients living in resource‐limited settings and reduce public health‐care costs. Further global testing of educational materials such as the one developed by Tosin et al.^1^ is warranted.

## Author Roles

(1) Research project: A. Conception, B. Organization, C. Execution; (2) Statistical analysis: A. Design, B. Execution, C. Review and critique; (3) Manuscript preparation: A. Writing of the first draft, B. Review and critique.

F.M.G.: 1A, 1B, 1C, 2A, 2B, 2C, 3A, 3B

A.F.S.S.: 2C, 3B

D.T.S.: 2C, 3B

P.R.P.B.: 2C, 3B

L.S.‐M.: 1A, 1B, 1C, 2A, 2B, 2C, 3A, 3B

## Disclosures

**Ethical Compliance Statement:** We confirm that we have read the journal's position on issues involved in ethical publication and affirm that this work is consistent with those guidelines. All subjects provided informed consent according to the protocol approved by the local ethics committee (project CAAE: 50374621.0.1001.5404, UNICAMP).

**Funding Sources and Conflicts of Interest:** F.M.G., D.T.S., A.F.S.S., P.R.P.B., L.S.‐M. The authors declare that there are no conflicts of interest relevant to this work.

**Financial Disclosures for the Previous 12 Months:** F.M.G. declares that there are no additional disclosures to report. D.T.S. reports receiving a research grant from The Michael J. Fox Foundation regarding a project on Parkinson's Disease Subtyping. A.F.S.S. declares receiving research grants from The Michael J. Fox Foundation, CNPq, and Fapergs. P.R.P.B. declares receiving research grants from The Michael J. Fox Foundation. L.S.‐M. declares employment at the Universidade Estadual de Campinas; research support from Samsung Eletronics, Ibrachina, and SAS Brasil; honoraria/small grants from AbbVie, Ipsen, FQM‐Roche, Teva, Zambon, Merz, and the International Parkinson and Movement Disorders Society.

## Data Availability

The data that support the findings of this study are openly available in REDU at https://redu.unicamp.br/dataset.xhtml?persistentId=doi:10.25824/redu/WMYTAG, reference number doi:10.25824/redu/WMYTAG.
